# Supplementary material for: Snow avalanche deaths in Switzerland from 1995 to 2014—Results of a nation-wide linkage study
Source: PLoS One. 2019 Dec 3;14(12):e0225735. doi: 10.1371/journal.pone.0225735 (PMC6890213; doi:10.1371/journal.pone.0225735)
Supplement: S1 Table — (PDF) [file pone.0225735.s001.pdf]

ICD 10 code	Explanation
W02	Fall involving ice-skates, skis, roller-skates or skateboards
W15	Fall from cliff
W17	Other fall from one level to another
W77	Threat to breathing due to cave-in, falling earth and other substances
X31	Exposure to excessive natural cold
X59	Exposure to unspecified factor causing fracture
Y86	Sequelae of other accidents
