# Supplementary material for: Snow avalanche deaths in Switzerland from 1995 to 2014—Results of a nation-wide linkage study
Source: PLoS One. 2019 Dec 3;14(12):e0225735. doi: 10.1371/journal.pone.0225735 (PMC6890213; doi:10.1371/journal.pone.0225735)
Supplement: S2 Table — (PDF) [file pone.0225735.s002.pdf]

Place of residence	Place of avalanche						Total
	Eastern Alps	*Southern Alps*	*Western Alps*	*Northern Alps*	*≤ 25km from the Northern Alps*	*> 25km from the Northern Alps*	
Eastern Alps	13	0	0	0	0	0	13
Southern Alps	0	1	1	1	0	0	3
Western Alps	2	0	33	0	0	0	35
Northern Alps	4	1	9	40	0	0	54
≤ 25km from the Northern Alps	19	1	10	13	1	0	44
> 25km from the Northern Alps	18	2	21	25	0	0	66
**Total**	56	5	74	79	1	0	215
