# Supplementary material for: Snow avalanche deaths in Switzerland from 1995 to 2014—Results of a nation-wide linkage study
Source: PLoS One. 2019 Dec 3;14(12):e0225735. doi: 10.1371/journal.pone.0225735 (PMC6890213; doi:10.1371/journal.pone.0225735)
Supplement: S3 Table — (PDF) [file pone.0225735.s003.pdf]

	All persons		15-39 yrs		40+ yrs	
**Characteristics**	**Hazard ratio**	**95% CI**	**Hazard ratio**	**95% CI**	**Hazard ratio**	**95% CI**
**Sex**		**p<0.001**		**p<0.001**		**p<0.001**
Male	1		1		1	
Female	0.195	0.125 - 0.304	0.213	0.111 - 0.406	0.181	0.098 - 0.335
**Region**		**p<0.001**		**p=0.001**		**p<0.001**
Eastern Alps	3.249	1.528 - 6.911	2.285	0.686 - 7.609	4.353	1.634 - 11.60
Southern Alps	0.473	0.115 - 1.953	0.468	0.063 - 3.466	0.486	0.065 - 3.624
Western Alps	5.392	3.075 - 9.454	4.146	1.779 - 9.662	6.824	3.194 - 14.58
Northern Alps	2.501	1.626 - 3.847	1.968	1.051 - 3.684	3.116	1.712 - 5.674
≤ 25km from the Northern Alps	1.027	0.652 - 1.616	0.806	0.415 - 1.566	1.282	0.684 - 2.402
> 25km from the Northern Alps	1		1		1	
