# Supplementary material for: Snow avalanche deaths in Switzerland from 1995 to 2014—Results of a nation-wide linkage study
Source: PLoS One. 2019 Dec 3;14(12):e0225735. doi: 10.1371/journal.pone.0225735 (PMC6890213; doi:10.1371/journal.pone.0225735)
Supplement: S4 Table — (PDF) [file pone.0225735.s004.pdf]

	All persons		15-39 yrs		40+ yrs	
**Characteristics**	**Hazard ratio**	**95% CI**	**Hazard ratio**	**95% CI**	**Hazard ratio**	**95% CI**
**Sex**		**p<0.001**		**p<0.001**		**p=0.013**
Male	1		1		1	
Female	0.095	0.038 - 0.239	0.077	0.024 - 0.247	0.153	0.035 - 0.679
**Region**		**p<0.001**		**p<0.001**		**p<0.001**
Eastern Alps	3.896	1.475 - 10.29	2.846	0.838 - 9.664	8.686	1.591 - 47.42
Southern Alps	0.479	0.065 - 3.557	0.606	0.081 - 4.538	0	0 - 0
Western Alps	7.875	3.966 - 15.63	6.597	2.963 - 14.69	13.72	3.432 - 54.86
Northern Alps	0.947	0.422 - 2.128	0.999	0.417 - 2.393	0.679	0.076 - 6.075
≤ 25km from the Northern Alps	0.596	0.282 - 1.259	0.433	0.172 - 1.091	1.349	0.337 - 5.394
> 25km from the Northern Alps	1		1		1	
